# Dupuytren’s disease cords are weakened ex vivo by injection of recombinant collagenase G – A proof-of-concept study

**DOI:** 10.1371/journal.pone.0359330

**Published:** 2026-09-24

**Authors:** Guy Rubin, David Rothem, Ran Tohar, Maayan Gal

**Affiliations:** 1 Orthopedic Department, Emek Medical Center, Afula, Israel; 2 Faculty of Medicine, Technion, Haifa, Israel; 3 Department of Oral Biology, Goldschleger School of Dental Medicine, Gray Faculty of Medical and Health Sciences, Tel Aviv University, Tel Aviv, Israel; Shanghai Jiao Tong University School of Medicine, CHINA

## Abstract

**Objectives:**

Dupuytren’s disease is a fibroproliferative disorder that is characterized by collagen-rich cords that cause progressive flexion contractures. Collagenase from *Clostridium histolyticum* is an established nonsurgical therapy. Herein, we evaluated whether a recombinant form of collagenase G (ColG), expressed in *Escherichia coli*, weakens Dupuytren’s cords ex vivo.

**Methods:**

Cords from Dupuytren’s disease patients were excised during surgery. Using a 30G needle, 0.5 mL of 4 mg/ml ColG or phosphate buffer Saline (PBS) was injected into the cord center. Samples were incubated in saline at 37 °C and mounted via size-1 Prolene Krackow sutures on an Instron tensile testing system. Force vs. displacement curves were recorded.

**Results:**

Six cords from five patients were tested (ColG n = 4; PBS n = 2). Representative force–displacement curves showed a reduced slope and lower peak force prior to rupture in ColG-treated samples compared with vehicle. The mean maximum rupture force was lower with ColG than with PBS (0.047 kN vs 0.069 kN), corresponding to ~32% reduction in the force required to tear the cord in this series.

**Conclusions:**

Recombinant ColG weakens the tensile strength of Dupuytren’s cords ex vivo, consistent with potent collagenolytic activity. These proof-of-concept pilot data support further studies to define dose–response, optimize formulation and exposure time, and evaluate safety and clinical performance relative to current collagenase-based treatments.

## Introduction

Dupuytren’s disease is a chronic, progressive fibroproliferative disorder [1,2]. The underlying pathophysiology involves abnormal proliferation of fibroblasts and myofibroblasts, excessive deposition of collagen, and disruption of normal extracellular matrix remodeling [3–5]. The disease is characterized by the formation of nodules and thickened cords in the palm, which can gradually contract and pull one or more fingers, most commonly the ring and little fingers, toward the palm, resulting in flexion contractures and impaired hand function. The condition can significantly limit daily activities such as grasping objects or placing the hand flat. An altered ratio of type I to type III collagen has been observed in the palmar fascia in this disease [6]. This process gives rise to a pathological cord that, over time, leads to digital flexion contracture. Although the epidemiology has not been comprehensively characterized, global prevalence is reported to range from 3% to 40%, with higher rates in white populations and in Northern Europe [7–9]. The disease primarily affects the ring and small fingers and involves the proximal interphalangeal (PIPJ) and metacarpophalangeal (MCPJ) joints [10,11]. Prevalence increases with age, and in men contractures tend to present approximately 10 years earlier than in women [12]. Contracture can be treated surgically by resecting (fasciectomy) or by dividing the cord (fasciotomy); however, this is not curative, as recurrence rates are high and range from 27% to 80% [13–16]. Surgery may also be accompanied by complications such as joint stiffness, nerve or vascular injury and infection [17].

Several non-surgical treatments have been attempted with limited success, including radiotherapy, dimethyl sulfoxide, physiotherapy, topical vitamins A and E, ultrasound, corticosteroid injection, 5-fluorouracil, and interferon-γ injection [18]. In addition, treatment of Dupuytren’s disease can be done via the application of collagenase enzymes that can digest the collagen polypeptide chain into short peptides. Of particular interest are bacterial collagenases that are considered important virulence factors due to their ability to efficiently digest collagen in the extracellular matrix (ECM) [19]. These enzymes assist in destroying extracellular structures, enabling efficient host colonization and penetration into anaerobic sites and promoting the spread of infection. By hydrolyzing collagen’s triple-helix structure, the enzyme weakens contracted cords, enabling clinicians to manually restore finger extension.

These collagenase properties have led to the development of a drug based on Collagenase G and H from *Clostridium histolyticum* as a groundbreaking non-surgical treatment for Dupuytren’s disease [20,21] and set the basis for the evaluation of the enzyme produced in *Escherichia coli* (*E. Coli*) in the current study. Indeed, multiple studies have reported encouraging outcomes with collagenase injection into the cord [22–25]. Based on these studies and following a successful phase 3 trial of collagenase injection for Dupuytren’s cords, the FDA approved the drug XIAFLEX for the treatment of Dupuytren’s disease [26]. By directly addressing the disease’s collagen-centric pathology, treatment of Dupuytren’s disease with collagenase represents a paradigm shift in Dupuytren’s management, balancing efficacy with reduced morbidity.

The clinical reliance on wild-type collagenases extracted from Clostridium histolyticum presents significant bottlenecks for both large-scale production and protein engineering. However, the feasibility of producing active recombinant collagenase G (ColG) in *E. coli* has been shown [20,21,27]. Importantly, this *E. coli*-derived enzyme retains robust in situ tissue degradation capabilities, as demonstrated by lowering the required physical force for exodontia via localized injection in the gums [28]. Moreover, beyond scalable production, recombinant expression has enabled the systematic screening and development of engineered protein variants with superior stability and accelerated enzymatic activity [29].

Building on the established tissue-degrading efficacy of ColG, the present study evaluates its proof-of-concept ability to digest pathological cords extracted from patients with Dupuytren’s disease. To this end, we quantified the biomechanical force required to rupture the fibrous cords following treatment with either the recombinant enzyme ColG or a vehicle control.

## Methods

### Ethics approval

The research was approved by the local Institutional Helsinki Committee (EMC-21–0174). All patients signed an informed consent before enrolling in the study.

### Enzyme expression and purification

Expression and purification of ColG was done in a similar way as previously described [27–30]. Briefly, the catalytic domain of collagenase G (ColG, residues 119–1118) was expressed using a recombinant bacterial system. Competent E. coli BL21(DE3) cells were transformed with the target plasmid and selected on ampicillin-agar plates. Seed cultures were grown in Luria-Bertani (LB) medium containing 100 μg/ml ampicillin. Upon the culture reaching an optical density (OD600) of 0.8, expression was initiated through induction with 1 mM IPTG for an additional 16 hours. The biomass was harvested via centrifugation and resuspended in lysis buffer (50 mM NaPi, 300 mM NaCl, 10 mM imidazole, pH 8.0) for sonication. The resulting homogenate was cleared at 10,000 g to isolate the soluble protein fraction. This supernatant was then loaded onto a nickel HisTrap FF column (Cytiva, USA) for affinity-based purification. To ensure high purity, the resin-bound protein underwent a rigorous three-stage washing by: 50 mM NaPi, 300 mM NaCl, 40 mM imidazole (pH 8.0); High-Salt Wash: 50 mM NaPi, 1 M NaCl, 10 mM imidazole (pH 8.0) to disrupt non-specific ionic interactions and 50 mM NaPi, 300 mM NaCl, 20% glycerol (pH 8.0), to wash non-specific binders. The target enzyme was subsequently recovered using an elution buffer enriched with 300 mM imidazole. Final processing involved concentration through a 30-kDa molecular weight cut-off (MWCO) Amicon Ultra-15 centrifugal filter (Merck, USA), followed by a buffer exchange into PBS via dialysis for downstream applications.

### Cord surgery and removal

Five patients were recruited for the study. During surgery, the cord was excised and, when feasible, divided into two segments. At each end of the cord, a size 1 Prolene suture was placed using a Krackow stitch. Using a 30G needle, 0.5 mL of the investigational substance was injected into the center of the cord. The cords were then immersed in a saline-filled container and immediately transported to the laboratory. The samples were incubated in saline at 37 °C for 2 hours. After incubation, the cords were attached by the sutures at their ends to a tensile testing device, and the rupture force was recorded in real-time. Within this pilot study, the absolute peak force (in Newtons) was utilized as the primary biomechanical endpoint.

### Force measurement

Continuous force-displacement data were acquired using an Instron Series 6800 universal testing system equipped with a 2-kilonewton (2 kN) loading cell (Instron Corp., Canton, MA, USA) as previously described [28,29]. Cord samples were subjected to steady, longitudinal tension at a controlled crosshead displacement rate of 10 mm/min. To accurately capture the failure mechanics, the pulling force was continuously logged at 10 Hz until macroscopic rupture of the cord.

### Statistical analysis

Statistical comparisons between the forces of the different samples were performed by an unpaired t-test using GraphPad Prism software. For statistical analysis, A p-value of < 0.05 was considered statistically significant (*p < 0.05).

## Results

**Fig 1:**
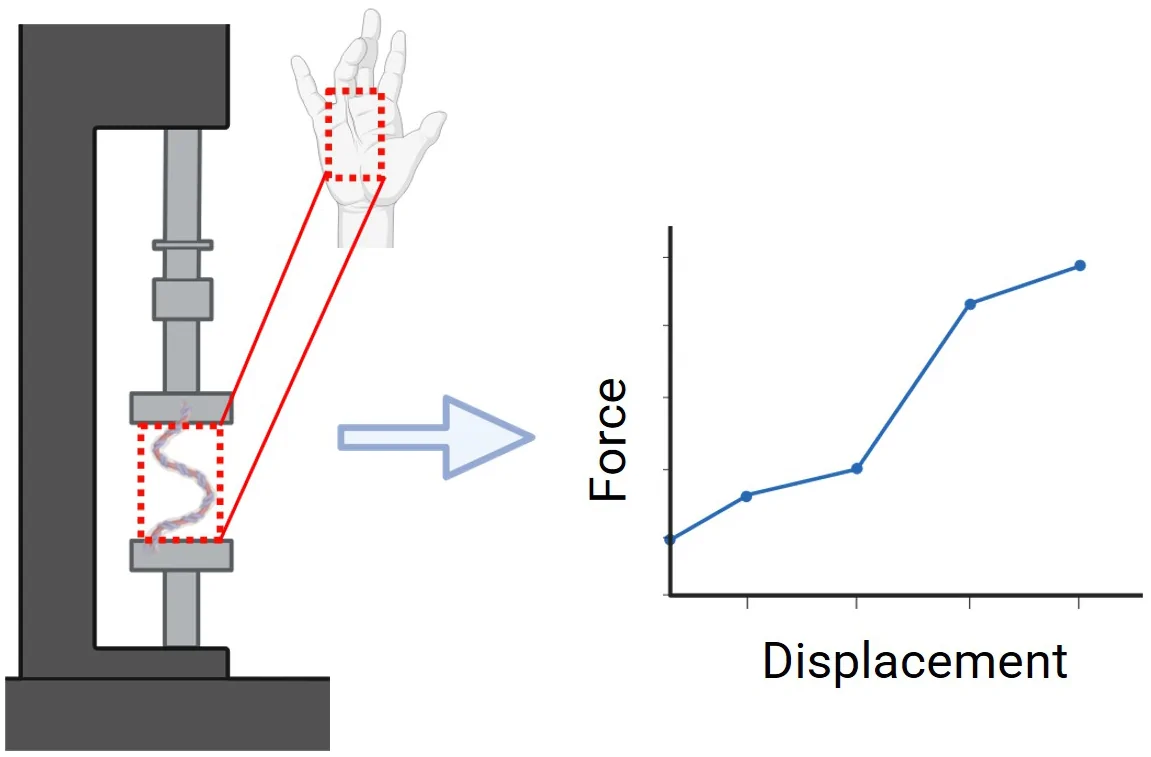
Ex vivo biomechanical tensile testing of Dupuytren’s cords. Schematic illustrating the experimental methodology for quantifying cord rupture force. Excised human Dupuytren’s cords were secured at both ends with surgical sutures, mounted between the grips of an Instron universal testing system, and subjected to a controlled vertical displacement. The resulting data allowed for the generation of continuous force-displacement curves to evaluate tissue integrity following enzymatic or vehicle treatment. Created with BioRender.com.

The experimental setup for tensile testing of the excised Dupuytren’s cords is shown in Fig 1. The excised Dupuytren’s cords were carefully prepared and securely tied at both ends with surgical sutures. Each cord was then mounted between the grips of the Instron device. Before preparing the cords for the loading machine, the cords were injected with ColG or PBS.

**Fig 2:**
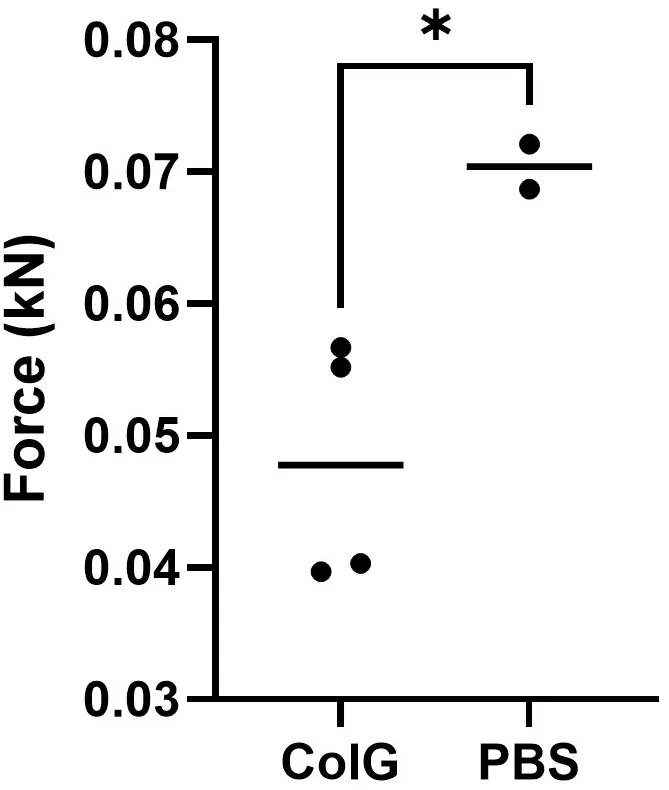
Recombinant ColG alters the biomechanical profile of Dupuytren’s cords. Representative force-displacement curves acquired during ex vivo tensile testing of cords treated with either PBS (vehicle control, left) or 4 mg/mL recombinant ColG (right). The PBS-treated cord exhibits a steep, progressive resistance to displacement. Conversely, the ColG-treated cord displays a reduced slope and a lower peak rupture force, functionally demonstrating the collagenolytic disruption of the tissue strength.

Fig 2 presents representative force versus displacement curves of two cords injected with PBS or ColG. The cord injected with vehicle requires a higher force to achieve elongation and ultimately to reach the point of tearing. In contrast, cords treated with ColG exhibit a reduced slope and a lower maximum force prior to rupture. This result demonstrates the potent collagenolytic activity of ColG and its ability to effectively disrupt the structural integrity of Dupuytren’s cords ex vivo. S1 Fig shows the individual force-displacement curves for all evaluated Dupuytren’s cords.

**Fig 3:**
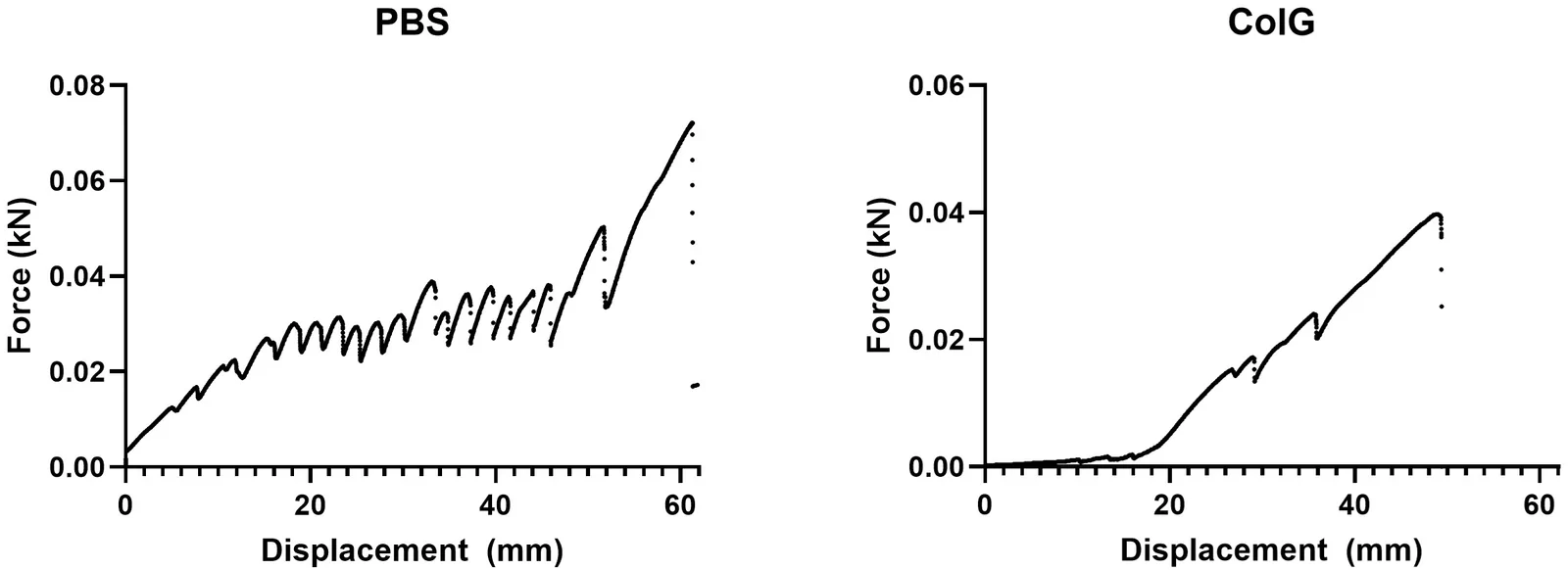
Quantification of biomechanical tissue disruption following enzymatic treatment. Scatter plot comparing the absolute peak rupture force (in kilonewtons, kN) of excised Dupuytren’s cords following incubation with either 4 mg/mL recombinant ColG (n = 4) or PBS vehicle control (n = 2). Cords treated with ColG required a lower average force to achieve structural failure compared to controls. Horizontal lines indicate the group mean. Statistical comparison was performed using an unpaired t-test, assuming normal Gaussian distribution and the same standard deviation (*p < 0.05).

Fig 3 shows the average maximum force required to rupture Dupuytren’s cords following injection of 4 mg/ml ColG or PBS. The single concentration was selected based on previous studies [28,29]. The data represent measurements from four cords injected with collagenase G and two cords injected with PBS. The results show that cords treated with ColG required a significantly lower average force to achieve tearing (0.047 kN) compared to those treated with PBS (0.069 kN).

## Discussion

While the injection of native Clostridium histolyticum collagenases has revolutionized the treatment of Dupuytren’s disease, their heterogeneous nature and the risk of unintended degradation of adjacent healthy collagenous tissues have driven the search for additional modalities with therapeutic benefits [31,32]. Among the microbial alternatives, collagenase derived from *Vibrio alginolyticus* (CVA) was developed. The purified, single-isoenzyme CVA has a highly selective collagenolytic profile, cleaving type I and III collagens while sparing other structural proteins within the ECM [33,34]. Beyond microbial sources, plant-derived proteases represent another translational approach. Bromelain, a proteolytic enzyme complex extracted from pineapple stems, has been investigated for its capacity to hydrolyze extracellular matrix components in various fibrotic conditions. However, its broader substrate specificity requires careful clinical consideration regarding localized tissue targeting [35]. While these natural extracts offer excellent baseline properties, they present inherent bottlenecks for large-scale, consistent manufacturing and structural optimization.

In this study, we provide ex vivo evidence that ColG, a recombinant collagenase enzyme expressed in *E. coli*, weakens the tensile strength of Dupuytren’s disease cords. Cords injected with ColG demonstrated a lower mean rupture force and flatter force–displacement curves compared with PBS controls, indicating effective enzymatic degradation of collagen within the pathological tissue. As noted, collagenase derived from Clostridium histolyticum is already an established nonsurgical therapy for Dupuytren’s contracture, with demonstrated efficacy in clinical practice. However, the currently available preparation is derived directly from the bacterial host and has limitations related to production, cost, and clinical response variability. Recombinant enzyme production offers several important benefits over natural extraction. Proteins can be manufactured at scale with greater purity and reproducibility, avoiding batch-to-batch variability. Furthermore, recombinant technology enables protein engineering, allowing tailoring of enzymatic activity, substrate specificity, and pharmacological properties. For Dupuytren’s disease, this may translate into enzymes with optimized activity that reduce the required dose, improve the precision of cord degradation, and minimize adverse effects such as skin tears or off-target proteolysis. Our ex vivo findings are consistent with prior reports in other tissues, such as dental models, where ColG reduced the mechanical force required for tissue separation. Together, these data strengthen the rationale for ColG as a versatile, recombinant alternative to native bacterial collagenases.

Our data demonstrate that recombinant *E. coli*–expressed ColG reduces the mechanical strength of Dupuytren’s cords ex vivo, consistent with potent collagenolytic activity in other models [28,29]. However, a major limitation of the present study is the low sample size of only six excised cords, and the evaluation of a single enzyme concentration. Given this small cohort, any statistical inferences drawn must be interpreted with caution. Furthermore, while ex vivo tensile testing is highly useful, it cannot fully replicate the dynamic clinical environment where enzyme distribution, tissue perfusion, and host immune responses significantly influence both efficacy and safety. Consequently, this investigation is most accurately characterized as a proof-of-concept and feasibility study. An additional limitation is the reliance on a biomechanical endpoint (rupture force). Future investigations must incorporate quantitative histological analyses and biochemical degradation assays to microscopically evaluate the enzymatic digestion and lifetime. Additionally, ex vivo models cannot account for variable enzyme distribution or the reproducibility of intralesional injections within dense fibrotic tissue, contributing to the inherent biomechanical variability observed between individual human specimens.

Despite these limitations, our initial findings successfully establish the biomechanical rationale for this engineered enzymatic approach. Future research must focus on defining optimal dosing and exposure times and directly comparing this recombinant variant with the native collagenase G currently used in the clinic. Ultimately, advancing this platform will require comprehensive in vivo preclinical and clinical trials to rigorously assess safety, immunogenicity, long-term durability of the correction, and patient-reported outcomes.

## Supporting information

S1 Fig Individual force-displacement curves for all evaluated Dupuytren’s cord specimens. Continuous force (kN) versus displacement (mm) data are presented for each of the six excised human cords tested in this proof-of-concept study. The top row displays the individual mechanical loading profiles for the four cords treated with 4 mg/mL recombinant ColG. The bottom row displays the loading profiles for the two control cords treated with the PBS vehicle. Each curve traces the tissue’s resistance to steady longitudinal tension up to the point of ultimate macroscopic rupture. These raw data illustrate the inherent biomechanical variability across independent human tissue samples and represent the complete dataset utilized to generate the representative curves in Fig 2 and the summary statistics in Fig 3.
(JPG)
